# Le syndrome d'encéphalopathie postérieure réversible chez un garçon sous dialyse péritonéale

**DOI:** 10.11604/pamj.2015.22.287.8041

**Published:** 2015-11-24

**Authors:** Manel Jellouli, Tahar Gargah

**Affiliations:** 1 Service de pédiatrie, Hôpital Charles Nicolle, Tunis, Tunisie

**Keywords:** Hypertension artérielle, convulsion, insuffisance rénale, hypertension, convulsion, renal failure

## Image en medicine

Le syndrome d'encéphalopathie postérieure réversible (SEPR) est une entité rare, d’étiopathogénie inconnue; le diagnostic est radio-clinique qui associe des signes neurologiques avec des anomalies radiologiques cérébrales bilatérales classiquement réversibles. La principale cause est l'hypertension artérielle sévère. MA âgé de 5 ans est suivi pour insuffisance rénale terminale (IRT) sous dialyse péritonéale automatisée. L’étiologie de son IRT est un syndrome hémolytique et urémique atypique avec la présence d'anticorps anti-facteur H au bilan immunologique. Le patient a présenté 1 an après la découverte de sa pathologie un état de mal convulsif. L'examen trouvait après la résolution des crises, un enfant conscient, une hypertension artérielle sévère à 190/110 mmHg. Il ne présentait pas de déficit sensitvo-moteur. L'hypertension artérielle était équilibrée par du nicardipine, captopril et prazosine. L'imagerie cérébrale par résonnance magnétique a montré des anomalies du signal bilatérales asymétriques de la substance blanche profonde en regard de deux carrefours ventriculaires cadrant avec une encéphalopathie hypertensive. Une IRM de contrôle à 6 mois d'intervalle a montré une régression nette des anomalies. Le diagnostic de SEPR est ainsi retenu.

**Figure 1:**
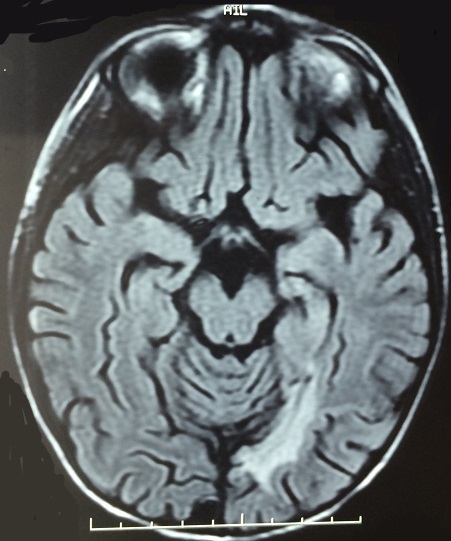
IRM cérébrale montrant la présence des anomalies de signal de l’étage sus tentoriel en hypersignal pouvant cadrer vu le contexte à un syndrome d'encéphalopathie postérieure réversible

